# Low-Frequency Intermittent Hypoxia Promotes Subcutaneous Adipogenic Differentiation

**DOI:** 10.1155/2018/4501757

**Published:** 2018-03-12

**Authors:** Yan Wang, Judith C. W. Mak, Mary Y. K. Lee, Aimin Xu, Mary S. M. Ip

**Affiliations:** ^1^Department of Medicine, Li Ka Shing Faculty of Medicine, Pokfulam, The University of Hong Kong, Hong Kong; ^2^Department of Pharmacology & Pharmacy, Li Ka Shing Faculty of Medicine, The University of Hong Kong, Pokfulam, Hong Kong; ^3^Research Centre of Heart, Brain, Hormone and Healthy Aging, Li Ka Shing Faculty of Medicine, The University of Hong Kong, Pokfulam, Hong Kong; ^4^State Key Laboratory of Pharmaceutical Biotechnology, Li Ka Shing Faculty of Medicine, The University of Hong Kong, Pokfulam, Hong Kong

## Abstract

Obstructive sleep apnea (OSA), characterized by intermittent hypoxia (IH), is associated with obesity and metabolic disorders. The mass and function of adipose tissue are largely dependent on adipogenesis. The impact of low-frequency IH on adipogenesis is unknown. Sprague-Dawley rats were subjected to IH (4 min for 10% O_2_ and 2 min for 21% O_2_) or intermittent normoxia (IN) for 6 weeks. The degree of adipogenic differentiation was evaluated by adipogenic transcriptional factors, adipocyte-specific proteins, and oily droplet production in both subcutaneous adipose tissue (SAT) and visceral adipose tissue (VAT). Upregulation of proadipogenic markers (CEBP*α*, PPAR*γ*, and FABP4) and downregulation of antiadipogenic markers CHOP in line with smaller size of adipocytes were found in IH-exposed SAT. In vitro experiments using human preadipocytes (HPAs) of subcutaneous lineage during differentiation phase, subjected to IH (1% O_2_ for 10 min and 21% O_2_ for 5 min; 5% CO_2_) or IN treatment, were done to investigate the insulin-like growth factor 1 receptor (IGF-1R)/Akt pathway in adipogenesis. IH promoted the accumulation of oily droplets and adipogenesis-associated markers. IGF-1R kinase inhibitor NVP-AEW541 attenuated the proadipogenic role in IH-exposed HPAs. In summary, relatively low frequency of IH may enhance adipogenesis preferentially in SAT.

## 1. Introduction

Intermittent hypoxia (IH), a characteristic feature of obstructive sleep apnea (OSA), is regarded as a pathogenic factor in OSA-related morbidities, including cardiovascular and metabolic disorders. Obesity, in particular visceral obesity, is a well-established factor contributing to the development of metabolic syndrome or various cardiometabolic diseases such as type 2 diabetes mellitus (DM) or hypertension [1–3]. Increasing evidence suggests that OSA may contribute towards obesity-induced metabolic disorders [4, 5].

Adipose tissue not only serves as an organ to control energy balance by storing and mobilizing triglycerides but also actively exerts profound impact on glucose metabolism, immunologic response, inflammatory response, and angiogenesis [6]. Various fat depots in the body are known to have different metabolism, and their excessive accumulation would predispose to different detrimental effects [7]. Adipose tissue accumulation is caused by enlargement of existing adipocytes (hypertrophy), generation of new adipocytes (hyperplasia), or both. Adipogenesis (adipogenic differentiation) is the process resulting in adipocyte hyperplasia. Adipocytes have a finite storage capacity, and once existing cells have reached that limit, new adipocyte formation is required to prevent lipid deposition in the liver, muscle, or other inappropriate locations. Most studies support a reduction of adipogenesis in the obese state [8].

Intermittent hypoxia, in various experimental models, can trigger both detrimental and beneficial effects on multiple body systems [9]. The features of IH profile (nadir hypoxia level and hypoxia episodes per hour) determine the outcomes, which would also differ in various organs/tissues. While detrimental effects of severe IH have been consistently demonstrated, the impact of lesser degrees of IH is not well characterized. Experimental data has demonstrated that IH of relatively low cycle numbers (5–15 episodes/hour) and less profound hypoxia (nadir of 9–16% inspired O_2_) may lead to beneficial rather than detrimental effects on the cardiovascular and metabolic systems [10–13]. The adverse effect of severe IH profile on adipose tissue and adipocyte dysfunction has also been investigated extensively [14–16], but relatively little is known about the impact of IH, especially of mild or modest degree, on adipogenesis.

We hypothesize that low frequency of IH may alter adipogenic differentiation in a depot-specific manner, through modulation of adipogenic transcriptional factors and/or adipogenic extracellular factors. Using the *in vivo* rat model of IH exposure, the effect of low-frequency IH on adipocyte differentiation status in VAT and SAT and the associated mechanisms were investigated. The extracellular signaling pathways and intracellular transcriptional factors for adipogenesis were further investigated in an *in vitro* IH-exposed subcutaneous adipocyte model.

## 2. Materials and Methods

### 2.1. *In Vivo* Intermittent Hypoxia- (IH-) Exposed Rat Model

Twelve healthy male Sprague-Dawley (SD) rats (~200 g; six-week old) were randomly divided into intermittent normoxia (IN) and IH groups. Rats were fed with standard chow diet. One rat from the IH group died during the course of study period. Treatments of IN and IH were simultaneously performed in 2 identical chambers (Oxycycler model A84; BioSpherix, Redfield, NY, USA) for 6 hours daily, during 9:00 am to 3:00 pm, for 6 weeks. The O_2_ concentration in the chamber was continuously measured by an O_2_ analyzer during the exposure (see Supplementary Figure S1). The profile of IH was designed as approximately 240 seconds (for 10% O_2_) and 120 seconds (for 21% O_2_). For IN, the period of hypoxic (10% O_2_) gas supply was replaced by air (21% O_2_) while keeping other chamber conditions the same. After 6-weeks IN or IH exposure, rats were sacrificed with overdose of sodium pentobarbital anesthesia (100 mg/kg, i.p.). Epididymal adipose tissue and inguinal adipose tissue were isolated to represent visceral (VAT) and subcutaneous (SAT) adipose tissues, respectively [17, 18]. Isolated tissues were snap-frozen and stored at −70°C for the measurements of mRNA and protein expressions for adipogenic transcriptional factors and adipogenic extracellular factors. Arterial blood was obtained via cardiac puncture. Rat serum was prepared by centrifugation at 2200 ×g for 10 minutes for measurement of metabolic parameters such as triglyceride, total cholesterol, glucose, and free fatty acid levels. All animal procedures conformed to the guidelines from Directive 2010/63/EU of the European Parliament. The experiment was approved by the Committee on the Use of Live Animal in Teaching and Research (CULATR number 2371–11) of The University of Hong Kong.

### 2.2. Adipose Tissue Morphometry

Epididymal adipose tissue (VAT) and inguinal adipose tissue (SAT) were collected in 10% buffered formalin, fixed overnight, and embedded in paraffin. Hematoxylin and eosin staining was used for adipocyte morphometry. Images were taken using Nikon Eclipse Ni-U microscope ×20 objective (Nikon Instruments Inc., Melville, NY, USA).

### 2.3. Adipogenic Differentiation in an *In Vitro* IH-Exposed Cell Model

Human primary subcutaneous preadipocytes (HPAs; ScienCell Research Laboratories, Carlsbad, CA, USA) were cultured in growth medium (Dulbecco's modified eagle's medium (DMEM) plus 20% fetal bovine serum (FBS)). After reaching 70–80% confluence, adipogenic differentiation was induced. HPAs underwent 6 differentiation cycles into mature adipocytes. Each differentiation cycle consisted of 3 days of differentiation period (DMEM/10% FBS with additional 10 *μ*g/ml insulin (Sigma, St. Louis, MO), 100 *μ*M indomethacin, 0.5 mM 3-isobutyl-1-methylxanthine, and 1 *μ*M dexamethasone (Sigma, St. Louis, MO, USA)) and 2 days of maintenance period (DMEM/10% FBS supplementation with 10 *μ*g/ml insulin). During each 3-day differentiation period, HPAs were subjected to IH exposure (1% for 10 min and 21% for 5 min per cycle; 5% CO_2_) or IN exposure (21% O_2_ and 5% CO_2_) in the chambers (Oxycycler model A42; BioSpherix, Redfield, NY, USA). The degree of differentiation was investigated using Oil Red O staining at every two cycles. After every cycle of differentiation, the adipocyte-derived conditioned media after IH or IN exposure were collected, sterile-filtered, and frozen at −70°C for future use. Total RNA and proteins were extracted from cells at every two cycles to detect differentiation-associated markers and transcriptional factors.

To investigate the role of IGF-1R/Akt signaling in IH-regulated adipogenesis, HPAs and differentiated HPAs were subjected to IH exposure for 96 cycles with the stimulation of 100 *μ*g/ml insulin (Sigma) in either growth medium (for HPAs) or maintenance medium (for differentiated HPAs). In addition, HPAs were incubated with a selective IGF-1R kinase inhibitor NVP-AEW541 [19] (0.1 *μ*mol/l, Cayman Chemicals, MI, USA) for 30 min before undergoing differentiation for 12 consecutive days.

### 2.4. Real-Time Reverse Transcription Polymerase Chain Reaction (RT-PCR) Analysis

For quantitative PCR (qPCR), total RNA was isolated from VAT, SAT, and HPAs using Trizol (Invitrogen, Life Technologies). Reverse transcription was performed using 1 *μ*g of total RNA with Qiagen RT kit (Qiagen, Life Technologies) according to the manufacturer's instructions. Primers for FABP4, GLUT4, PPAR*γ*, CEBP*α*, CEBP*δ*, and GAPDH were designed by Primer 3 (BioTools, University of Massachusetts Medical School, USA) and synthesized by IDT (Integrated DNA Technologies, Singapore). The sequences for primers are listed in Table 1. Amplification of target was carried out with Power SYBR Green PCR Mix (Applied Biosystems, Life Technologies Inc., California, USA) using StepOnePlus Real-Time PCR System (Applied Biosystems, Life Technologies Inc., California, USA). The cycle threshold value for amplification was normalized using glyceraldehyde 3-phosphate dehydrogenase (GAPDH) as the reference gene, and the data were analyzed using the ∆Ct method. Data represent the mean ± SEM of independent experiments.

### 2.5. Western Blot Analysis

The SAT, VAT, HPAs, and differentiated HPAs were lysed in tissue/cell lysis/extraction reagent (Thermo Scientific, Waltham, MA, USA) supplemented with 1/100 (*v/v*) protease inhibitor cocktail (Thermo Scientific, Waltham, MA, USA). The protein concentrations of tissue/cell lysates were determined using the Bradford assay. Protein extracts from tissue (30 *μ*g) or cells (15 *μ*g) were separated on a 10% acrylamide gel and transferred to a nitrocellulose membrane (100 V, 2 h). For tissue lysates, blots were probed for FABP4 (1 : 200, Santa Cruz, Texas, USA), CHOP (1 : 1000, Cell Signaling, MA, USA), insulin growth factor 1-receptor (IGF-1R, 1 : 1000, Cell Signaling, MA, USA), and GAPDH (1 : 1000, Santa Cruz, Texas, USA). For cell lysates, blots were stained for IGF-1R (1 : 1000, Cell Signaling, MA, USA), PPAR*γ* (1 : 1000, Cell Signaling, MA, USA), p-Akt (Ser 473) (1 : 1000 Cell Signaling, Danvers, MA, USA), Akt (1 : 1000, Cell Signaling, Danvers, MA, USA), and GAPDH (1 : 1000, Santa Cruz, Texas, USA). Densitometric analysis of the bands was performed with ImageJ (1.45v, National Institutes of Health, USA) to determine the respective protein expression levels.

### 2.6. Measurement of Adiponectin from Conditioned Media of Adipose Tissue

Approximately 400 mg fresh SAT and VAT was isolated and chopped into pieces and then incubated with 1 ml medium (DMEM/F12 plus 10% FBS) for 24 hours. After incubation, conditioned media were centrifuged at 500*g* for 5 min and stored at −70°C. The levels of adiponectin being released in conditioned media were analyzed using commercial ELISA kits (eBioscience, San Diego, CA, USA) according to the manufacturer's instructions.

### 2.7. Determination of Lipid Accumulation in HPAs by Oil Red O Staining

HPA cells were stained with Oil Red O dye to determine the lipid content of adipocytes. Cells were washed with ice-cold PBS 2 times and then fixed with 10% formalin for 30 minutes at room temperature. After discarding the fixative, the cells were rinsed with 60% isopropanol. Cells were finally stained with Oil Red O working solution (Sigma, St. Louis, MO; 5% in isopropanol, freshly diluted 2 : 3 with water) for 30 min and washed with 60% isopropanol. The stained cells were photographed under a microscope. The quantification of Oil Red O was performed by the area of Oil Red O staining above a constant area using ImageJ (1.45v, National Institutes of Health, USA) to calculate the percentage of area stained positive for Oil Red O.

### 2.8. Releases of Inflammation-Associated Mediators from Cultured HPAs

Conditioned media were collected from HPAs at the end of each maintenance period of adipogenic differentiation. The releases of IL-6 and MCP-1 in conditioned media were analyzed using commercial ELISA kits, respectively (eBioscience, San Diego, CA, USA) according to the manufacturer's instructions.

### 2.9. Statistical Analysis

Data were expressed as mean ± SEM from at least 4 independent cell culture experiments or experimental animals. Data from the IH and IN groups were compared by Student's *t*-test.

The variance equality was analyzed for all comparisons. If variance equality is not met, the unpaired *t*-test with Welch's correction was used. All statistical analyses were performed via GraphPad Prism 5.0 (GraphPad Software Inc., San Diego, CA, USA). A *P* value (two-sided) <0.05 was considered statistically significant.

## 3. Results

### 3.1. Alteration in Adipogenic Markers and Regulators from Depot-Specific Adipose Tissues after IH Exposure

**Figure 1:**
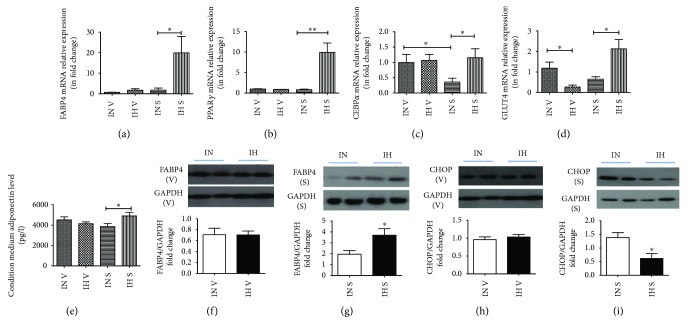
Intermittent hypoxia (IH) accelerated subcutaneous adipose tissue (SAT) but not visceral adipose tissue (VAT) adipogenesis. The gene expression levels of adipogenic markers and regulators in depot-specific adipose tissue after 6-week IH exposure. The mRNA expressions of FABP4 (a), PPAR*γ* (b), CEBP*α* (c), and GLUT4 (d) were measured by real-time PCR. The adiponectin (e) levels released into conditioned medium were measured with ELISA. Protein expressions of adipogenic markers and regulators in depot-specific adipose tissue after IH exposure (f–i). The protein expressions of FABP4 (f and g) and CHOP (h and i) in VAT and SAT were detected by Western blots. *N* = 5–6. Bars: mean ± SEM. ^∗^ *P* < 0.05 and ^∗∗^ *P* < 0.01.

After IH exposure, there was a nonsignificantly lower weight gain at 6 weeks in the rats exposed to IH, compared to the IN group (see Supplementary Figure S2). In IN condition, there were no significant difference of mRNA expressions of FABP4, PPAR*γ*, and GLUT4 between VAT and SAT, but that of CEBP*α* in SAT was significantly lower than in VAT. IH exposure caused significant elevation of FABP4 (*P* = 0.0387; *t* = 3.031; df = 4), PPAR*γ* (*P* = 0.0025; *t* = 6.765; df = 4), CEBP*α* (*P* = 0.0239; *t* = 2.872; df = 7), and GLUT4 (*P* = 0.0190; *t* = 3.806; df = 4) mRNA expression in SAT but not in VAT (Figures 1(a)–1(d)). The mRNA expression of GLUT4 (*P* = 0.0355; *t* = 2.857; df = 5) was significantly suppressed in IH-exposed VAT (Figure 1(d)). There was no difference in mRNA expression of CEBP*δ* in both SAT and VAT (data not shown). IH exposure also facilitated the release of adiponectin (*P* = 0.0360; *t* = 2.249; df = 20) from SAT rather than VAT (Figure 1(e)). Consistent with the mRNA expression, IH exposure significantly increased the protein expression of FABP4 in SAT (*P* = 0.0121; *t* = 3.230; df = 8) but not in VAT (*P* = 0.9759; *t* = 0.03108; df = 9) (Figures 1(f) and 1(g)). On the other hand, IH exposure caused significant downregulation of protein expression of C/EBP homologous protein (CHOP), an inducible inhibitor of adipogenic differentiation in response to metabolic stress [20], in SAT (*P* = 0.0287; *t* = 2.663; df = 8) but not in VAT (*P* = 0.5097; *t* = 0.6866; df = 9) (Figures 1(h) and 1(i)).

### 3.2. Regulation of IGF-1R in Depot-Specific Adipose Tissue after IH Exposure

**Figure 2:**
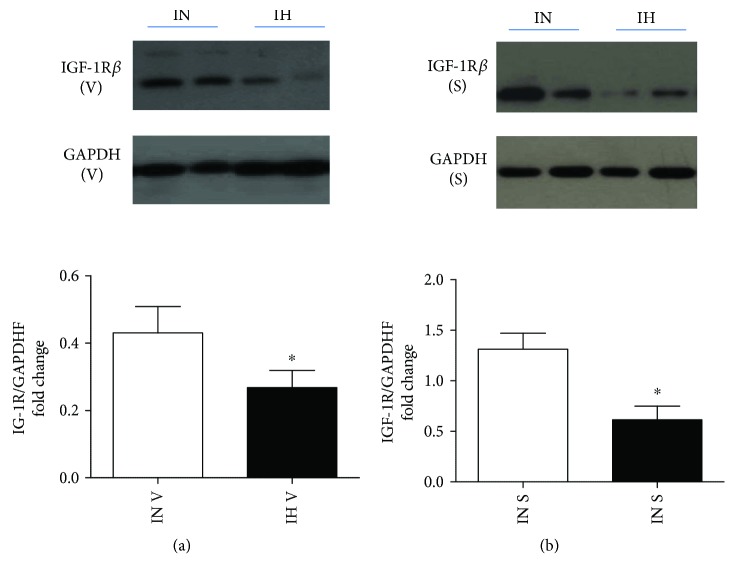
Protein expression of insulin growth factor-1 receptor (IGF-1R) in both subcutaneous adipose tissue (SAT) and visceral adipose tissue (VAT) after intermittent hypoxia (IH) exposure. The protein expression of IGF-1R was detected in SAT (a) and VAT (b) via Western blots. *N* = 5–6. Bars: mean ± SEM. ^∗^ *P* < 0.05.

IGF-1 receptor (IGF-1R) has high affinity for insulin and is a critical mediator in insulin-induced metabolic effects. After IH exposure, the protein expressions of IGF-1R*β* was downregulated significantly in both SAT (*P* = 0.0147; *t* = 3.098; df = 8) and VAT (*P* = 0.0205; *t* = 2.806; df = 9) (Figures 2(a) and 2(b)).

### 3.3. Adipose Tissue Morphometry

**Figure 3:**
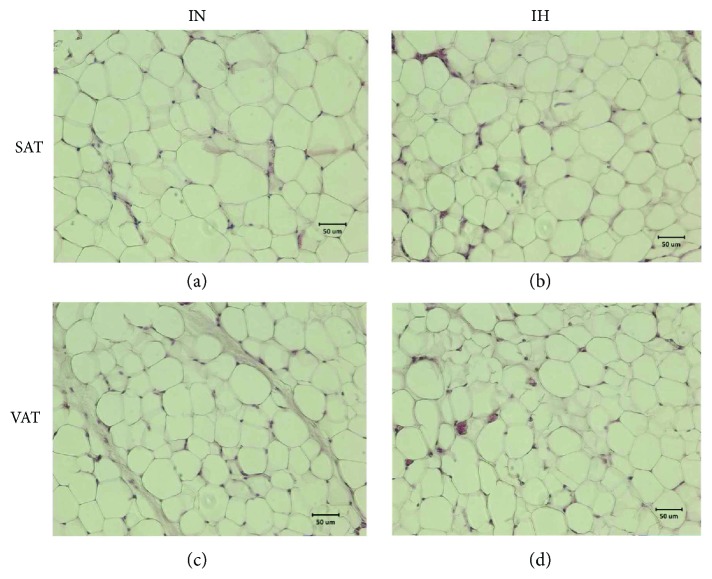
Intermittent hypoxia (IH) caused smaller size of adipocytes with an increase in number in subcutaneous adipose tissue (SAT) but not in visceral adipose tissue (VAT). (a–d) Photomicrographs of adipocytes from rat adipose tissue (hematoxylin-eosin staining, ×20 magnification): (a) and (c) SAT and VAT of IN group, respectively; (b) and (d) SAT and VAT of IH group, respectively, SAT but not VAT with smaller size of adipocytes and the elevation of number of adipocytes, suggesting the presence of hyperplasia.

The IH group showed smaller size of adipocytes with an increase in number compared to the IN group in SAT (Figures 3(a) and 3(b)) but not in VAT (Figures 3(c) and 3(d)), suggesting the presence of hyperplasia.

### 3.4. Acceleration of Adipogenic Differentiation in HPAs after IH Exposure

**Figure 4:**
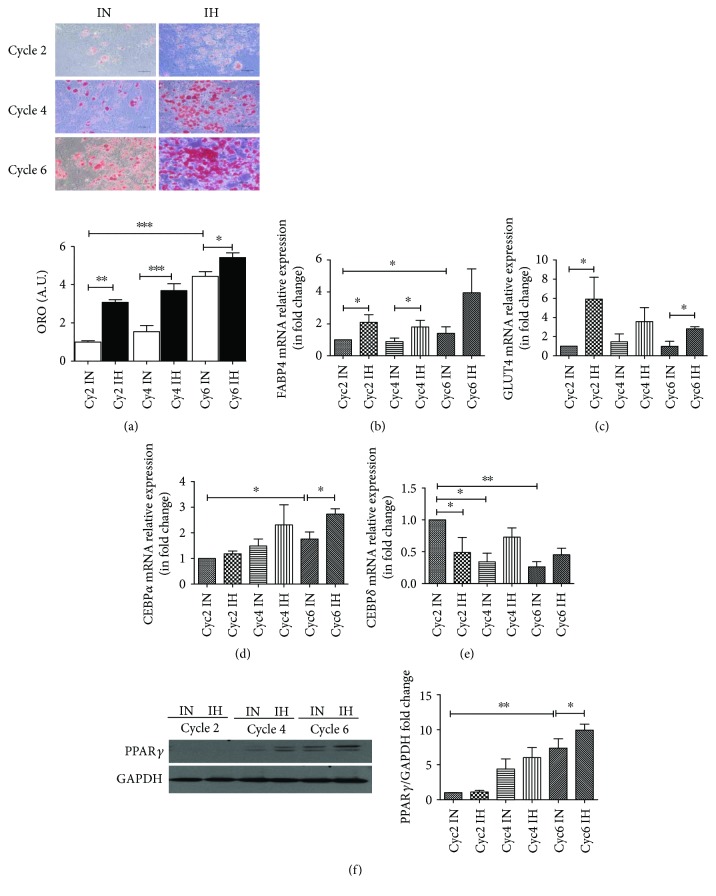
Intermittent hypoxia (IH) exposure promoted human primary subcutaneous preadipocyte (HPA) adipogenesis. Oil Red O staining was carried out for the identification of differentiation (a) (*N* = 4). One differentiated cycle was defined as 3-day differentiation and 2-day maintenance. The expressions of differentiation-associated markers during the conversion of HPAs into adipocytes (b–f). The mRNA expressions of FABP4 (b), GLUT4 (c), CEBP*α* (d), and CEBP*δ* (e) were quantified using real-time PCR. The protein expression of PPAR*γ* (f) was measured via Western blots. The results were expressed as mean ± SEM as fold change with respect to control (IN) at cycle 2. (*N* = 5). Bars: mean ± SEM. ^∗^ *P* < 0.05, ^∗∗^ *P* < 0.01, and ^∗∗∗^ *P* < 0.001.

After induction of adipogenic differentiation without or with IH exposure, HPAs went through obvious morphological changes from fibroblast-like to round cells containing lipid droplets (Cyc2 IN and IH: *P* = 0.0002, *t* = 13.15, and df = 4; Cyc4 IN and IH: *P* = 0.0117, *t* = 4.400, and df = 4; Cyc6 IN and IH: *P* = 0.0461, *t* = 2.857, and df = 4; Cyc2 IN and Cyc6 IN: *P* = 0.0002, *t* = 13.33, and df = 4) (Figure 4(a)). Using Oil Red O staining, a gradual increase in the lipid production of HPAs was observed during progressive cycles of differentiation. Acceleration of adipogenic differentiation was observed under IH exposure, as shown by the elevation of lipid accumulation in comparison to IN exposure at the same cycle. In comparison to IN exposure, IH induced elevated expressions of FABP4 (Cyc2 IN and IH: *P* = 0.0384, *t* = 2.791, and df = 5; Cyc4 IN and IH: *P* = 0.0317, *t* = 2.599, and df = 8; Cyc6 IN and IH: *P* = 0.0407, *t* = 2.743, and df = 5; Cyc2 IN and Cyc6 IN: *P* = 0.0407, *t* = 2.743, and df = 5) and GLUT4 (Cyc2 IN and IH: *P* = 0.0209, *t* = 3.694, and df = 4; Cyc6 IN and IH: *P* = 0.0454, *t* = 2.519, and df = 6) mRNA over different cycles of adipogenic differentiation (Figures 4(b) and 4(c)). CCAAT/enhancer-binding protein (C/EBP) *α* was significantly promoted by IH exposure at cycle 6 (*P* = 0.0248, *t* = 2.975, and df = 6) (Figure 4(d)). In addition, CEBP*δ* plays a crucial role in the activation of adipogenesis. A significant reduction of CEBP*δ* mRNA expression was observed at increasing cycles of differentiation under IN condition (Cyc2 IN and Cyc4 IN: *P* = 0.0064, *t* = 6.852, and df = 3; Cyc2 IN and Cyc6 IN: *P* = 0.0028, *t* = 9.086, and df = 3) (Figure 4(e)). IH exposure prevented the accelerated reduction of CEBP*δ* throughout the induction of adipogenic differentiation (Cyc2 IN and Cyc2 IH: *P* = 0.0215, *t* = 4.423, and df = 3; Cyc4 IN and Cyc4 IH: *P* = 0.0331, *t* = 2.755, and df = 6; Cyc6 IN and Cyc6 IH: *P* = 0.1974, *t* = 1.449, and df = 6) (Figure 4(e)). In addition, IH exposure, compared to IN, enhanced the protein expression of PPAR*γ* during adipogenic differentiation (Cyc6 IN and Cyc6 IH: *P* = 0.0418, *t* = 2.580, and df = 6) (Figure 4(f)).

### 3.5. The Involvement of IGF-1R/Akt Signaling Pathway in IH-Regulated HPA Adipogenic Differentiation

**Figure 5:**
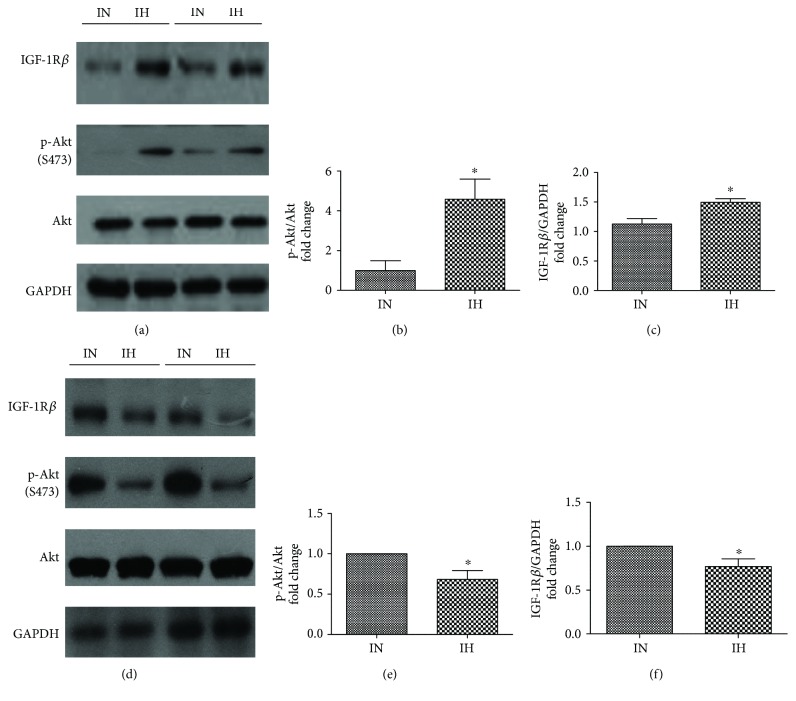
Insulin growth factor-1 receptor (IGF-1R)/Akt signaling pathway in human primary subcutaneous preadipocytes (HPAs) and differentiated HPAs after intermittent normoxia (IN) or intermittent hypoxia (IH) exposure. The protein expressions of IGF-1R*β* and phosphorylated protein p-Akt (Ser 473) in HPAs (a–c) and in differentiated HPAs (d–f) were examined by Western blots. *N* = 4. Bars: mean ± SEM. ^∗^ *P* < 0.05.

**Figure 6:**
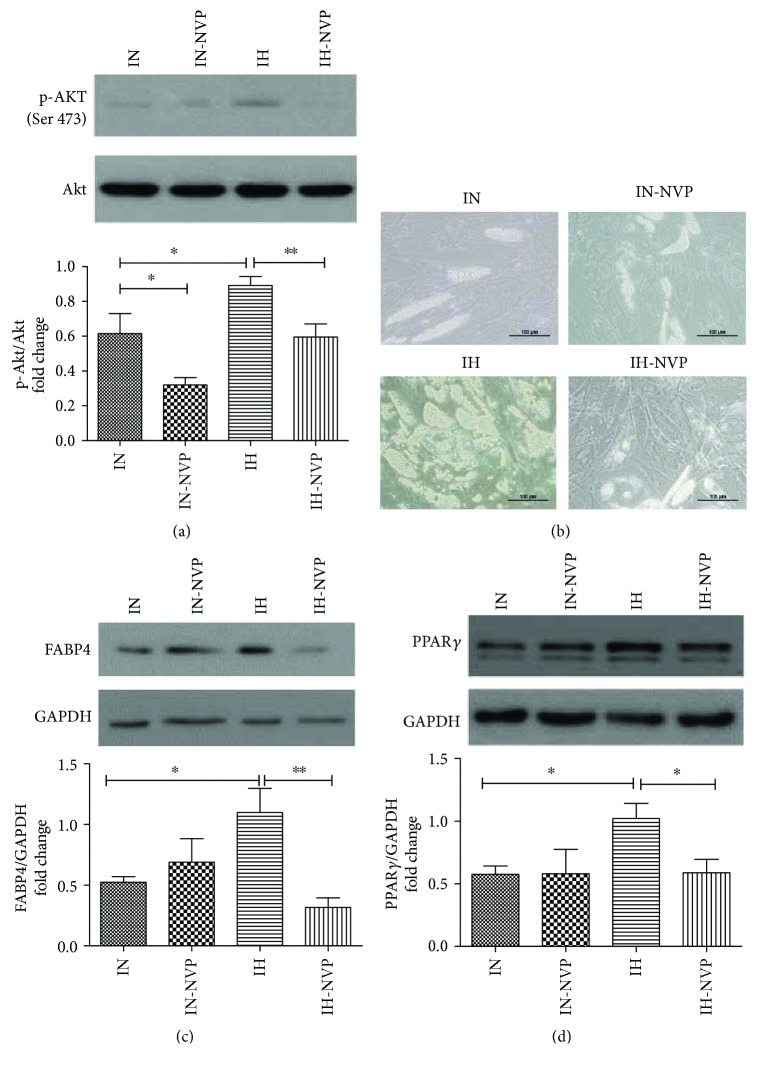
The Akt signaling and adipogenic differentiation in the absence or presence of a selective insulin growth factor-1 receptor (IGF-1R) kinase inhibitor NVP-AEW541. The protein expressions of p-Akt (Ser 473) and total Akt were detected by Western blots (a). The phase-contrast picture showed oily droplets after adipogenic differentiation of HPAs in IN and IH condition (b). The protein expressions of adipogenic markers FABP4 (c) and PPAR*γ* (d) were measured by Western blots. The results were expressed as mean ± SEM as fold change with respect to control (IN). *N* = 5. Bars: mean ± SEM. ^∗^ *P* < 0.05 and ^∗∗^ *P* < 0.01.

IGF-1R/Akt signaling pathway was investigated in HPAs (i.e., preadipocytes) and differentiated HPAs (i.e., adipocytes) by Western blot analysis. After 96 cycles of IH exposure, protein expression of IGF-1R*β* was upregulated significantly in HPAs (preadipocytes) compared to IN exposure (*P* = 0.0158, *t* = 3.328, and df = 6) (Figures 5(a) and 5(c)). IH exposure also caused enhancement of protein expression of p-Akt (Ser 473) with no change in total Akt, which may drive the subsequent differentiation process in HPAs (preadipocytes) (*P* = 0.0188, *t* = 3.190, and df = 6) (Figures 5(a) and 5(b)). On the contrary, the exposure of differentiated HPAs to IH for 96 cycles led to significant downregulation of IGF-1R*β* (*P* = 0.0392, *t* = 2.628, and df = 6) protein expression and p-Akt (Ser 473) (*P* = 0.0447, *t* = 2.887, and df = 4) protein expression (Figures 5(d)–5(f)). A marked inhibitory effect of NVP-AEW541 on p-Akt (Ser 473) was observed (IN and IH: *P* = 0.0382, *t* = 2.478, and df = 8; IN and IN-NVP: *P* = 0.0546, *t* = 2.382, and df = 6; IH and IH-NVP: *P* = 0.0088, *t* = 3.331, and df = 9) (Figure 6(a)). In spite of this, NVP-AEW541 obviously decelerated the production of oily droplets under IH exposure but not under IN exposure (Figure 6(b)). In agreement with the oily droplets, the protein expressions of differentiation-associated markers FABP4 (IN and IH: *P* = 0.0489, *t* = 2.799, and df = 4; IH and IH-NVP: *P* = 0.0036, *t* = 3.898, and df = 9) and PPAR*γ* (IN and IH: *P* = 0.0126, *t* = 3.202, and df = 8; IH and IH-NVP: *P* = 0.0359, *t* = 2.590, and df = 7) were also significantly downregulated in IH-exposed HPAs in the presence of NVP-AEW541 (Figures 6(c) and 6(d)). There was no obvious difference exhibited between IN treatment with/without NVP-AEW 541.

### 3.6. IH Induced Dysregulation of Inflammation during Adipogenic Differentiation of HPAs

**Figure 7:**
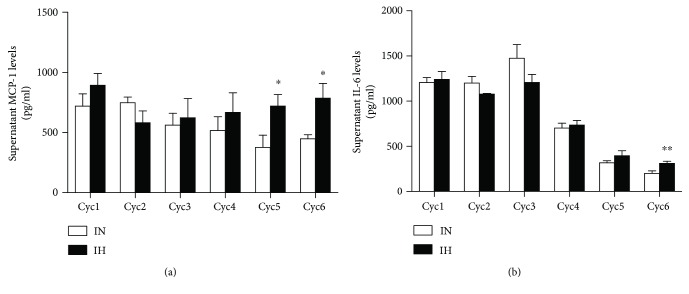
The release of proinflammatory mediators during human primary subcutaneous preadipocyte (HPA) differential process. Levels of proinflammatory mediators MCP-1 (a) and IL-6 (b) in conditioned media were detected by ELISA. *N* = 6. Bars: mean ± SEM. ^∗^ *P* < 0.05 and ^∗∗^ *P* < 0.01.

With induction of adipogenic differentiation, trends of reduction in basal levels of MCP-1 and IL-6 release and increment in basal level of adiponectin release were observed. IH induced significant elevations of MCP-1 (Cyc5 IN and IH: *P* = 0.0328, *t* = 2.475, and df = 10; Cyc6 IN and IH: *P* = 0.0446, *t* = 2.665, and df = 5) (Figure 7(a)) and IL-6 (Cyc6 IN and IH: *P* = 0.0098, *t* = 3.181, and df = 10) (Figure 7(b)) in HPAs after adipogenic differentiation, suggesting that IH induced a proinflammatory response in the mature adipocytes.

## 4. Discussion

This study provided evidence that daily IH exposure of relatively low frequency (10 events/hour) for 6 hours over 6 weeks accelerated adipogenesis of SAT but not of VAT in a lean rat model. In line with the *in vivo* findings, IH induced acceleration of differentiation of human preadipocytes of subcutaneous lineage (HPAs) *in vitro*. Mechanistically, IH potentiated transcriptional cascades CEBP*α*, CEBP*δ*, PPAR*γ*, and IGF-1/Akt signaling pathway as well as proinflammatory mediators in SAT or subcutaneous HPAs.

In recent years, the potential role of OSA in the pathogenesis of metabolic abnormalities has been extensively investigated both in clinical studies and in animal models [21, 22]. As adipose tissue exerts important endocrine functions in metabolic regulation, the complex relationship between OSA, adipose tissue dysfunction, and other metabolic derangements is noted. Intermittent hypoxia, a hallmark pathophysiologic feature in OSA, has been reported to aggravate adipose tissue dysfunction and metabolic disorders [14, 15], while data on the impact of IH on adipogenesis is limited.

In comparison to subcutaneous obesity, visceral obesity tends to be associated with OSA-associated insulin resistance, dyslipidemia, and glucose intolerance [23]. Increased deposition of fat in the visceral compartment may occur as an unfavorable outcome consequent upon saturation of SAT to store fat [24]. Unlike adiposity accumulation induced by over nutrition, our *in vivo* data showed that IH at this relatively low frequency upregulated adipogenesis in SAT but not in VAT, in agreement with their respective adipocyte morphometry. These findings implied that adipogenic progenitor cells from different depots responded differently to the same IH stimulus. In line with the *in vivo* upregulation of FABP4 and GLUT4 expression in SAT, IH also stimulated upregulation of FABP4 and GLUT4 in HPAs *in vitro*, supporting the regulation of adipogenesis by IH in subcutaneous fat depot.

The two fat depots, VAT and SAT, have different cellular characteristics and origins, [25, 26], which may contribute to the differential effect of IH. Compared to VAT, SAT contains higher capacity for preadipocyte proliferation and differentiation [27–29], implying that adipogenic ability may be a dominant response to stress stimuli in SAT. In this study, the morphological changes induced by IH were consistent with enhanced adipogenesis with smaller adipocytes in SAT but not in VAT. Furthermore, cellular response elicited by IH would likely be determined by the severity of the stimulus. Our *in vivo* model exposed mice to IH 10 cycles/hour, while a previous study which showed visceral adipose tissue dysmetabolism used an IH regimen of 20 cycles/hour [30]. It has been reported that low abdominal subcutaneous preadipocyte adipogenesis is associated with visceral obesity and a dysmetabolic state [31]. Hence, it is tempting to speculate that subcutaneous adipogenesis seen in the current study indicates a mechanism by which the subcutaneous fat depot is acting as a metabolic buffer in the face of IH challenge.

There is a recent hypothesis that mild OSA may have a “protective” effect on metabolic hemostasis, probably due to preconditioning to hypoxia [32–34]. Subcutaneous fat is the largest adipose depot in the human body for storage of excess lipids, and if SAT undergoes hyperplasia with generation of smaller and more functional adipocytes, this added functional capacity may attenuate or reduce the risk of metabolic diseases [35]. Recent animal studies proposed a therapeutic role of transplantation of subcutaneous fat in alleviating type 2 diabetes mellitus, obesity, and insulin resistance [36–38]. In addition, the removal of visceral fat and replacement with subcutaneous fat could alleviate or prevent metabolic dysregulation [39]. In obesity, subcutaneous adipocyte hyperplasia precedes adipocyte hypotrophy [40]. In this IH-exposed rat model, there was a significant elevation of serum lipid markers such as triglyceride (TG) and free fatty acid (FFA) (see Supplementary Figure S3), similar to a previous study [41]. The enhanced adipogenic capacity of subcutaneous preadipocytes after IH exposure may be a defensive response to produce more mature adipocytes for the safe storage of lipids, reducing the chance of ectopic deposition of lipids which triggers dysmetabolism. Notwithstanding the enhanced adipogenesis seen, serum triglyceride remained elevated, suggesting still inadequate capacity.

Differentiation ability of adipocytes is regulated by various transcription factors [42]. The expression of PPAR*γ* is required to induce 3T3L1 differentiation [43], and we demonstrated upregulation of PPARγ during adipogenic differentiation with or without IH exposure. C/EBP*α* induces many adipocyte genes directly, and *in vivo* studies have indicated an important role for this factor in the development of adipose tissue [44]. In our *in vivo* model, upregulation of C/EBP*α* was demonstrated in SAT but not in VAT. In addition, the SAT of IH-exposed rats showed a significant reduction of CHOP, an inducible inhibitor of adipocyte differentiation [20], in comparison with that of IN-exposed rat, providing another possible mechanism responsible for IH-induced acceleration of subcutaneous adipogenesis. Besides PPAR*γ* and CEBP*α*, CEBP*δ* is crucial in the early stage of adipogenesis as it could activate mitotic clonal expansion (MCE) which is an indispensable factor for the induction of preadipocytes into differentiated programming [45]. In agreement with previous studies, a remarkable reduction of CEBP*δ* was observed in the control (IN) differentiable HPAs after initiation of adipogenesis. IH exposure significantly mitigated the reduction of CEBP*δ*, suggesting that IH potentiated more preadipocytes into differentiation and further extended the initial stage of adipogenesis.

Apart from various transcriptional factors, IGF-1R/Akt signaling also functions for the transduction of differential signals. IGF-1 and insulin are essential factors for the initiation of adipocyte differentiation [46, 47]. The effect of insulin on adipogenic differentiation is due to its binding with IGF-1 receptors (IGF-1Rs), which are more abundant in preadipocytes compared to mature adipocytes [48, 49]. Our in vitro results indicated that IH significantly upregulated the expression of IGF-1Rs along with increased phosphorylation of Akt in HPAs (preadipocytes), suggesting the involvement of the IGF-1R/Akt pathway in IH-driven adipogenesis. In support, the selective inhibitor of IGF-1R significantly suppressed IH-facilitated HPA differentiation. However, it is unexpected that a similar effect did not exist in IN-exposed HPAs at the same concentration of IGF-1R inhibitor, which may be attributed to robustly compensatory adipogenic pathways in normal preadipocytes. Besides, the expressions of IGF-1R pathway were significantly suppressed in both IH-exposed differentiated HPAs (mature adipocytes) and adipose tissue (SAT and VAT). The possible reason is that IGF-1R is associated with insulin resistance in mature adipocytes, which accounted for approximately 80% in adipose tissue [48, 50]. As IH has been shown to induce insulin resistance [47], in agreement with an elevation of serum glucose level in IH-exposed rats (Supplementary Figure S3), the findings that downregulated expressions of IGF-1R pathway in both IH-exposed differentiated HPAs (mature adipocytes) and adipose tissue (SAT and VAT) provide novel mechanistic insight into the relationship between IH and insulin resistance of mature adipocytes.

In addition to extracellular signals and the transcriptional cascades, proinflammatory signaling in the adipocyte has recently been identified as an essential step for adipose tissue expansion [51]. Our *in vitro* study showed that as HPA cells matured in normoxic condition, less IL-6 and MCP were secreted compared to the earlier stages of cell proliferation and maturation, suggesting that proinflammatory capacity might be more prominent at the beginning of physiologic adipogenic differentiation. However, when the cells reached maturity, IH exposure compared to IN led to an increase in the inflammation profile, consistent with our previous *in vivo* data using the same IH profile [52].

There are several limitations in the current study. Firstly, our findings, being a snapshot at a specific time point of low-frequency IH exposure in a lean rat model, cannot distinguish if the enhanced subcutaneous adipogenesis is acting as a “metabolic sink” or it is an early phase of detrimental adipose tissue accumulation nor can the findings be generalized to that in the obese state. Secondly, the *in vitro* IH model as used in the current study, or in most other *in-vitro* IH studies in the literature, has inherent limitations. Oxygenation sensed by the cells would be derived via oxygen diffusion from the gas in the culture chamber into the culture medium, a process that highly depends on the rate of diffusion and equilibrium between gas/liquid, which is far less efficient compared to the biologic system in the animal model. Thus, the real IH pattern experienced by the cells in the *in vitro* system would not be comparable to that seen in the *in vivo* model and the cellular events cannot be precisely correlated with the oxygen concentration or pattern to which the cells were exposed [53]. Finally, our study focused on adipocytes, while adipose tissues comprise of both adipocytes and macrophages which are of vital importance in fat tissue metabolism. In obese or hypoxia state, adipose tissue inflammation is accompanied by an imbalance in the ratio of M1/M2 macrophages, with the enhancement of M1 proinflammatory macrophages and the downregulation of M2 anti-inflammatory macrophages [54]. It has been reported that IH exposure (20 cycles/hour, nadir of FiO2: 6.4% for 8 weeks) promoted M1/M2 macrophage polarization in VAT and AT surrounding tumors [55]. It is still unknown whether macrophage infiltration plays any role in the adipogenesis of IH-exposed preadipocytes in lean state, and further studies would be needed to investigate this specific aspect.

**Figure 8:**
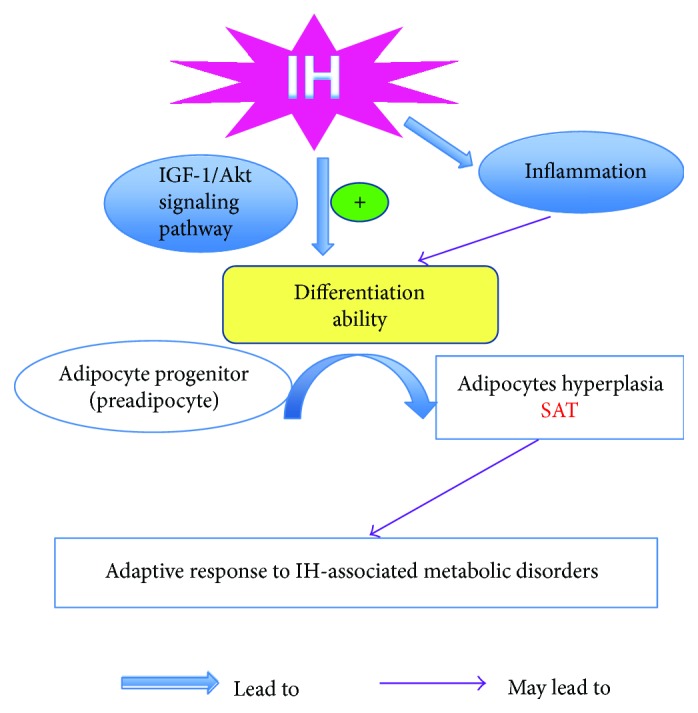
Schematic diagram summarizing the role of intermittent hypoxia (IH) in adipogenesis in *in vivo* and *in vitro* model. Low-frequency IH exposure could activate insulin growth factor-1 receptor (IGF-1R)/Akt to promote adipogenic differentiation of subcutaneous preadipocytes. In addition, the enhanced inflammation elicited by IH exposure could be another possible mechanism responsible for intensified adipogenesis of subcutaneous adiposity. The accelerative subcutaneous adiposity may be a compensatory response to IH-stimulated metabolic disorders.

In summary, this study demonstrated that IH promoted subcutaneous adipogenesis *in vivo* in the lean rat model and this was confirmed in an *in vitro* model of human subcutaneous preadipocytes. The IH-induced subcutaneous adipogenesis involved transcriptional factors including CEBP*α*, CEBP*δ*, PPAR*γ*, and IGF-1R/Akt signaling pathway (Figure 8). As subcutaneous fat is the lesser metabolically harmful adipose depot, our findings of enhanced subcutaneous adipogenesis is further hypothesized to be part of a homeostatic response to IH challenges, in particular when the IH is of mild degree. In the face of the growing epidemic of obesity and associated OSA which comprise of a wide range of severities, it is of tremendous clinical relevance to understand the role of IH on evolution of adipose tissue expansion and distribution. Our current finding is novel and warrants further research, which may shed light on whether the body's intrinsic responses may be harnessed to mitigate adverse sequelae.

## Figures and Tables

**Table 1:** Primer sequences used in this study.

Gene names	Forward primer (5′–3′)	Reverse primer (5′–3′)
hFABP4	GAAGTAGGAGTGGGCTTTGC	ATTCCTGGCCCAGTATGAAG
hGLUT4	GATAGGCTCCGAAGATGGG	CCAGCCACGTCTCATTGTAG
hCEBP*δ*	CAGAAGTTGGTGGAGCTGTC	TTACCGGCAGTCTGCTGTC
hCEBP*α*	CAAGAAGTCGGTGGACAAGA	GGTCATTGTCACTGGTCAGC
hGAPDH	GTCAAGGCTGAGAACGGGAA	AAATGAGCCCCAGCCTTCTC
Rat FABP4	AAATGAGCCCCAGCCTTCTC	TGCAAATTTCAGTCCAGGGC
Rat CEBP*α*	GGCCAAGAAGTCGGTGGA	TTGACCAAGGAGCTCTCAGG
Rat GLUT4	GCCATCGTCATTGGCATTCT	CGCTTTAGACTCTTTCGGGC
Rat PPAR*γ*	AGGGGATCTTGACAGGAAA	CGAAACTGGCACCCTTGAAA
Rat GAPDH	TCATCAACGGGAAACCCATCAC	ACGCCAGTAGACTCCACGACA

## Additional Points

*Statement of Significance.* Obstructive sleep apnea is strongly associated with metabolic disorders, and intermittent hypoxia from recurrent obstructed breathing may be a key pathogenetic factor in metabolic dysfunction. Adipose tissue plays a pivotal role in metabolic regulation, and its expansion is largely dependent on adipocyte differentiation (adipogenesis). We demonstrated that relatively low-frequency intermittent hypoxia in a lean mouse model promoted adipogenesis of subcutaneous but not visceral fat tissue, and the finding was corroborated by enhanced adipogenesis in human subcutaneous preadipocyte cultures. The novel findings suggest a differential response of subcutaneous and visceral fat depot towards this relatively milder degree of IH challenge. A deeper understanding of the underlying mechanisms by which OSA modulates adipose tissue metabolism would be critical in improving management strategy in sleep-disordered breathing which has a wide range of severities.

## Supplementary Materials

Supplementary Materials Supplementary Figure S1: (A) a recording of O_2_ concentration in the chamber (Oxycycler model A84; BioSpherix, Redfield, NY, USA). (B) A monitoring of O_2_ saturation (SpO_2_) in rat during IH10 exposure (10% O_2_ 4 min; 21% O_2_ 2 min). The range of readings was recorded using a noninvasive pulse oximeter for laboratory animals (Mouse Vent G500, Kent Scientific Corporation, Torrington, CT, USA) according to the manufacturer's instruction. Supplementary Figure S2: body weight (in g) over time in the IN and IH groups. There was no significant difference between groups. Supplementary Figure S3: serum levels of metabolic parameters in intermittent normoxia- (IN-) and intermittent hypoxia- (IH-) exposed rats. Triglyceride (TG) (A), free fatty acid (FFA) (B), and glucose (C) were determined via commercial kits. *N* = 5–6. Bars: mean ± SEM. ^∗^ *P* < 0.05.
