# Supplementary material for: Low-Frequency Intermittent Hypoxia Promotes Subcutaneous Adipogenic Differentiation
Source: Oxid Med Cell Longev. 2018 Mar 12;2018:4501757. doi: 10.1155/2018/4501757 (PMC5867560; doi:10.1155/2018/4501757)
Supplement: Supplementary Materials — Supplementary Figure S1: (A) a recording of O2 concentration in the chamber (Oxycycler model A84; BioSpherix, Redfield, NY, USA). (B) A monitoring of O2 saturation (SpO2) in rat during IH10 exposure (10% O2 4 min; 21% O2 2 min). The range of readings was recorded using a noninvasive pulse oximeter for laboratory animals (Mouse Vent G500, Kent Scientific Corporation, Torrington, CT, USA) according to the manufacturer's instruction. Supplementary Figure S2: body weight (in g) over time in the IN and IH groups. There was no significant difference between groups. Supplementary Figure S3: serum levels of metabolic parameters in intermittent normoxia- (IN-) and intermittent hypoxia- (IH-) exposed rats. Triglyceride (TG) (A), free fatty acid (FFA) (B), and glucose (C) were determined via commercial kits. N = 5–6. Bars: mean ± SEM. ∗ P < 0.05. [file 4501757.f1.docx]

**Supplementary Materials**

Low Frequency Intermittent Hypoxia Promotes Subcutaneous Adipogenic Differentiation

Yan Wang, PhD^1^, Judith C.W. Mak, PhD^1-3^, Mary Y.K. Lee, PhD^1,3^, Aimin Xu, PhD^1,2,4^, Mary S.M. Ip, MD^1,3^

*Departments of ^1^Medicine and ^2^Pharmacology & Pharmacy, Li Ka Shing Faculty of Medicine, The University of Hong Kong, Hong Kong;*

*^3^Research Centre of Heart, Brain, Hormone and Healthy Aging, Li Ka Shing Faculty of Medicine, The University of Hong Kong, Hong Kong;*

*^4^State Key Laboratory of Pharmaceutical Biotechnology and Department of Medicine, Li Ka Shing Faculty of Medicine, The University of Hong Kong, Hong Kong*

**Corresponding author:**

Professor Mary S. M. Ip,

Division of Respiratory Medicine, Department of Medicine, The University of Hong Kong, 4/F, Professorial Block, Queen Mary Hospital, Pokfulam, Hong Kong

Tel: (+852) 2255 4250

Fax: (+852) 2255 4604

E-mail: msmip@hku.hk

**Legends for Supplementary Figures**

**Supplementary figure S1:** (A) A recording of O_2_ concentration in the chamber (Oxycycler model A84; Biospherix, Redfield, NY, USA). (B) A monitoring of O_2_ saturation (SpO_2_) in rat during IH10 exposure (10% O_2_ 4 min; 21% O_2_ 2min). The range of readings was recorded using a noninvasive pulse oximeter for laboratory animals (Mouse Vent G500, Kent Scientific Corporation, Torrington, CT, USA) according to manufacturer’s instruction.

**Supplementary figure S2:** Body weight (in g) over time in IN and IH groups. There was no significant difference between groups.

**Supplementary figure S3:** Serum levels of metabolic parameters in intermittent normoxia (IN)- and intermittent hypoxia (IH)-exposed rats. Triglyceride (TG) (A), free fatty acid (FFA) (B), and glucose (C) were determined via commercial kits. N=5-6. Bars: mean ± SEM. **P*<0.05.


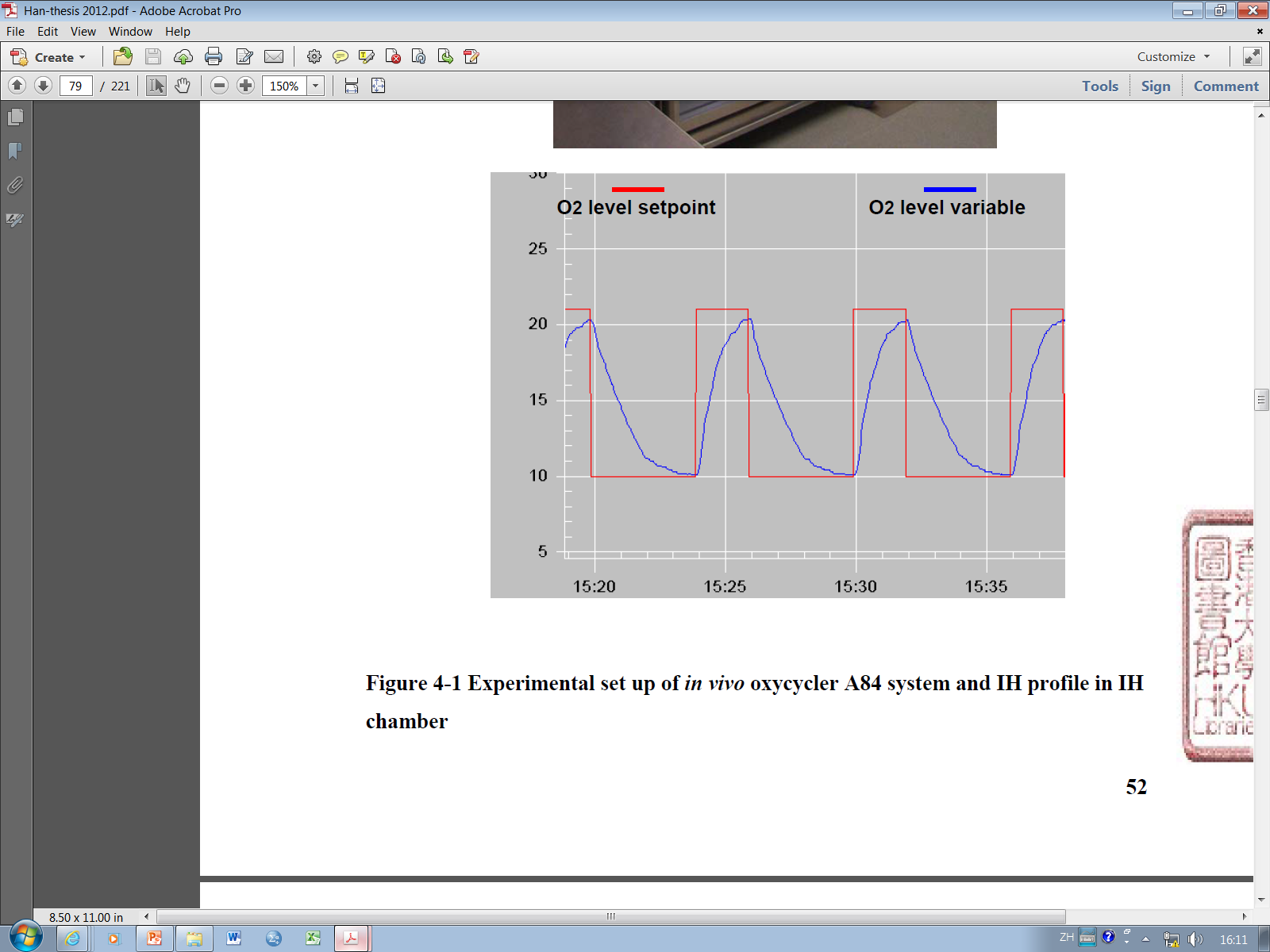

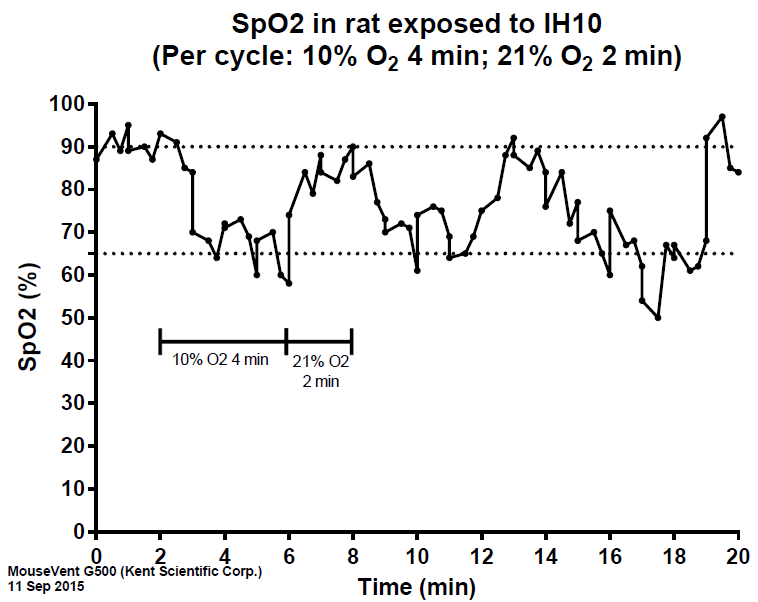
Supplementary figure S1

**A**

**B**

**6 minutes**

Supplementary figure S2


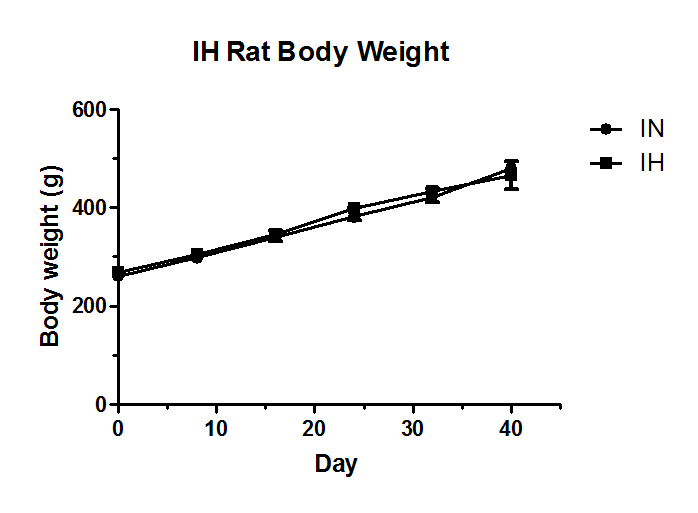

Supplementary figure S3

**A**

**B**

**C**
